# Efficacy and improvement of lipid profile after switching to rilpivirine in resource limited setting: real life clinical practice

**DOI:** 10.1186/s12981-019-0222-6

**Published:** 2019-04-05

**Authors:** Sivaporn Gatechompol, Anchalee Avihingsanon, Tanakorn Apornpong, Win Min Han, Stephen J. Kerr, Kiat Ruxrungtham

**Affiliations:** 1 HIV-NAT, Thai Red Cross AIDS Research Centre, 104 Ratchadamri Road, Pathumwan, Bangkok, 10330 Thailand; 2 Faculty of Medicine, Chulalongkorn University and King Chulalongkorn Memorial Hospital, Thai Red Cross Society, Rama 4 Road, Pathumwan, Bangkok, 10330 Thailand

**Keywords:** Switching, Rilpivirine, Resource limited setting, Dyslipidemia, HIV

## Abstract

**Background:**

Long-term success of cART is possible if the regimen is convenient and less-toxic. This study assessed the efficacy and safety of switching from a first-line NNRTI or boosted PI-based regimens to RPV-based regimens among virologically suppressed participants in resource-limited setting (RLS).

**Methods:**

This is a prospective cohort study. Participants with plasma HIV-RNA < 50 copies/mL receiving cART were switched from a PI- or NNRTI-based, to a RPV-based regimen between January 2011 and April 2018. The primary endpoint was the proportion of patients with plasma HIV-1 RNA level < 50 copies/mL after 12 months of RPV. The secondary endpoint was the virological response at 24 months and safety endpoint (change in lipid profiles and kidney function from baseline to 12 months).

**Results:**

A total of 320 participants were enrolled into the study. The rationale for switching to RPV was based on toxicity of the current regimen (57%) or desire to simplify cART (41%). Totally, 177 (55%) and 143 (45%) participants were on NNRTI and boosted PI, respectively, prior to switching to RPV. After 12 months, 298 (93%) participants maintained virological suppression. There were significant improvements in the lipid parameters: TC (− 21 (IQR − 47 to 1) mg/dL; p < 0.001), LDL (− 14 (IQR − 37 to 11) mg/dL; p < 0.001) and TG (− 22 (IQR − 74 to 10) mg/dL; p < 0.001). Also, there was a small but statistically significant decrease in eGFR (− 4.3 (IQR − 12 to 1.1) mL/min per 1.73m2; p < 0.001).

**Conclusions:**

In RLS where integrase inhibitors are not affordable, RPV-based regimens are a good alternative option for PLHIV who cannot tolerate first-line NNRTI or boosted PI regimen, without prior NNRTI/PI resistance.

*Trial registration* HIV-NAT 006 cohort, clinical trial number: NCT00411983

## Background

For the past decade, combined antiretroviral therapy (cART) has led to a marked reduction in mortality and morbidity among Human immunodeficiency virus (HIV)—infected participants worldwide [1, 2]. The participants on antiretroviral therapy can have life expectancy close to the general population [3]. However, life-long suppressive therapy is required. Currently, rapid cART initiation is recommended and thus, numbers of people living with HIV (PLHIV) on cART is on the rise [4]. Treatment fatigue and long-term adverse-effects.

(AE) of cART can negatively impact participant’s adherence as well as the quality of life. The US Department of Health and Human Services (DHHS) guidelines estimate that treatment-related side effects lead to regimen discontinuation in up to 10% of clinical trial participants [5]. Therefore, there is a need for further simple, efficacious, well tolerated, affordable antiretroviral therapy (ART) regimens in low-and middle-income countries (LMIC) where the burden of HIV is greatest. Rilpivirine (RPV) is a second-generation non-nucleoside reverse transcriptase inhibitor (NNRTI) that has recently been approved for the treatment of HIV-1 infection [6]. In the pooled ECHO and THRIVE studies, RPV demonstrated non-inferior efficacy in reducing HIV-1 RNA to < 50 copies/mL at weeks 48 and 96; it was well tolerated compared to efavirenz (EFV) when used for first-line treatment in participants with a baseline viral load below 100,000 copies/mL [7–9].

In the United States and Europe, a single tablet regimen (STR) composed of rilpivirine/emtricitabine/tenofovir disoproxil fumarate (RPV/FTC/TDF) is approved for use in HIV-1—infected, anti-retroviral-naïve individuals with baseline HIV-1 RNA ≤ 100,000 copies/mL and for PLHIV with suppressed viral load without known NNRTI, Tenofovir Disoproxil Fumarate (TDF) or emtricitabine (FTC) [5]. Since, no teratogenicity was reported [10], RPV might also be a good option in women of childbearing potential. Additionally, RPV concentration were above the protein-binding adjusted EC90 in most pregnant women [11]. As RPV use for ARV naïve patients is restricted to PLHIV with baseline HIV RNA < 100,000 copies/mL, and as HIV Ribonucleic acid (RNA) testing prior to ART initiation may not always be available in many LMIC, RPV based ART would be better suited for PLHIV on stable ART with suppressed VL.

RPV is well tolerated and convenient to take. It is also an attractive option for virologically suppressed treatment-experienced participants who are willing to switch their current treatment regimen to improve tolerability or convenience. However, RPV must be taken with a meal to enhance absorption and exposure [12] which may be cumbersome for some participants. Because RPV has to be taken with food, this may impact the efficacy of RPV in real clinical practice.

Previous studies have demonstrated that switching from ritonavir-boosted PI (PI/r) or NNRTI based regimen to RPV/FTC/TDF in virologically suppressed participants was effective and safe [13–15]. However, one observational study showed that at week 96, there were high rates of virological failure and treatment discontinuation because of the adverse events after switching to RPV based regimen [16].

There are a limited number of studies looking at switching to RPV-based regimens in real life clinical practice, especially in LMIC. Nowadays, there are new HIV regimens that have fewer side effects compared to the current regimens such as integrase inhibitors (INIs) which are expensive and are not available through the Thai National HIV program. Therefore, it is a challenge to manage participants who experience adverse effects or cannot tolerate 1st line NNRTI or protease inhibitor (PI) based regimens. As a result of this, we aimed to assess the efficacy and safety of switching from a first-line NNRTI or boosted PI-based regimens to RPV-based regimens among virologically suppressed Thai participants in real clinical practice.

## Methods

### Study design and population

From January 2011 to April 2018, we conducted a prospective cohort study at HIV-NAT, Thai Red Cross AIDS Research Centre (TRC-ARC), Bangkok, Thailand.

Participants without history of NNRTI failure with current plasma HIV-RNA < 50 copies/mL and receiving PI- or NNRTI-based regimens for more than 6 months were switched to RPV-based regimens; either with TDF + FTC, TDF + lamivudine (3TC) or abacavir (ABC) + 3TC. Participants were grouped according to their baseline regimens. Group 1 was composed of participants taking PI-based regimens before switching to RPV-based regimens; Group 2 was composed of participants taking NNRTI-based regimens before switching to RPV-based regimens. All participants were advised to take RPV with food.

At baseline, the following items were recorded: clinical characteristics, cART history, duration of HIV infection, duration of virological suppression before switching to RPV, comorbidities and reasons for switching to a RPV-based regimen. Baseline laboratory parameters were either collected at the time of switch or at the closest visit before the switch. Participants were asked to check viral load 3 to 6 months after switching to RPV regimen and then followed every 6 months for evaluate lipid profiles, kidney and liver function. All participants were advised to fast before blood chemistries were done. Data regarding AEs and treatment interruption were recorded when they were clinically observed.

The study was reviewed and approved by the institution’s review board. All participants voluntarily provided written informed consent prior to enrolling into the study.

### Efficacy and safety assessments

The primary objective of this study was to assess the virological response at 12 months after switching to a RPV-based regimen in virologically suppressed, HIV-1-infected participants who previously were on NNRTI or PI based regimens. Virological success was defined as participants with plasma HIV-1 RNA level was < 50 copies/mL at 12 months. Virological failure was defined as having a confirmed HIV-1 RNA level ≥ 50 copies/mL or an HIV-1 RNA level ≥ 50 copies/mL followed by RPV discontinuation.

Secondary end points included virological response at 24 months and the safety end point was defined as the change in lipid profiles such as total cholesterol (TC), high-density lipoprotein (HDL), low-density lipoprotein cholesterol (LDL) and triglycerides (TG). Kidney function was measured by estimated glomerular filtration rate using the CKD-EPI and liver function (ALT) was recorded.

### Statistical analyses

The proportion of participants with plasma HIV-1 RNA level < 50 copies/mL after 12 and 24 months of RPV treatment was evaluated using the HIV-1 RNA level at that month. Participants who discontinued RPV treatment, had virological failure or were lost to follow-up were classified as having unfavorable outcomes. The analysis was intent-to-treat. The difference in proportions with virological suppression at 12 and 24 months between NNRTI and boosted PI groups were compared using a Chi square test.

Sample size was based on the precision of the 95% CI around the assumed prevalence of failure after switch. We assumed the failure rate would be 5%; if 300 patients were enrolled, the 95% CI around a 5% failure would run from 2.9 to 8.1, a precision of approximately − 2 to + 3%, and an acceptable level of accuracy.

Mean (SD), median (IQR), and frequencies (%) were used to describe the participants’ characteristics in each study group. Chi square and student *t* test or Mann–Whitney U tests were used to formally compare categorical and continuous variables between the two groups (PI- and NNRTI-pre-treated groups), respectively.

Changes in CD4+ T-cell counts, estimated glomerular infiltration rate (eGFR), alanine aminotransferase (ALT), TC, TG, HDL and LDL from baseline to month 12 were analyzed using paired t-test or Wilcoxon signed-rank test whereas the difference of change between groups were used student t-test or Mann–Whitney U test.

## Results

Total of 362 participants were invited to the study but only 320 participants were enrolled. The reasons for not enrolling included having detectable viral load at baseline > 50 copies/mL (n = 22) and using previous regimens other than NNRTI and PI (n = 20). The enrollment period is between January 2011 and April 2017 but the follow up period up to April 2018. The majority of the participants were males (56%) and the median age was 46 (interquartile range (IQR) 41–50) years. Median duration of ART was 12 (IQR 8–16) years. Baseline median CD4 cell count was 674 (IQR 522–851) cells/mm^3^. Forty-five percent of all participants had viral load > 100,000 copies/mL before starting ART. TDF was used as Nucleoside reverse transcriptase inhibitors (NRTI) backbone component in 95% of participants and 6% of participants used ABC.

The rationale for switching to RPV was mainly due to toxic adverse-effects of the current regimen (57%) or desire to simplify cART (41%). Totally, 177 (55%) and 143 (45%) participants were on NNRTI and boosted PI, respectively, prior to a switch to RPV. The median duration of viral load suppression before switching to RPV was 8.6 (IQR 3.7–11.1) years. Median value of lipid profiles prior to switch were total cholesterol (TC) 210 (IQR 182–239) mg/dL; low-density lipoprotein cholesterol (LDL) 131 (IQR 107–153) mg/dL; high-density lipoprotein (HDL) 46 (IQR 38–53) mg/dL; and triglycerides (TG) 138 (IQR 94–206) mg/dL. Eighty-four participants (26.3%) took lipid lowering agent before the study entry and 29% of them discontinued lipid lowering agents within 12 months. The participants’ characteristics are shown in Table 1.

**Table 1:** Baseline characteristic of patients

Characteristics	Total (N = 320)	PI base (n = 143)	NNRTI base (n = 177)	p-value*
Age, years, median (IQR)	46 (41–50)	46 (41–50)	46 (41–50)	0.45
Male sex, n (%)	178 (55.63)	69 (48.25)	109 (61.58)	0.02
Baseline body weight, Kg, median (IQR)	61 (53–69)	61 (54–69)	61 (52–69)	0.83
Duration of HIV infection, year, median (IQR)	14 (11–18)	14 (11–19)	15 (11–18)	0.57
Duration of ART, years, median (IQR)	12 (8–16)	12 (9–17)	12 (5–16)	0.13
Duration of virological suppression, years, median (IQR)	8.6 (3.7–11.1)	8.6 (4.0–11.0)	8.7 (3.5–11.3)	0.90
Nadir CD4+ T-cell count, cells/μL, median (IQR)	210 (121–294)	203 (108–275)	221 (136–304)	0.16
Baseline CD4+ T-cell count, cells/μL, median (IQR)	674 (522–851)	696 (541–870)	649 (502–814)	0.09
Highest viral load before starting ART (n, %)				0.62
< 100,000 copies/mL	151 (54.71)	71 (56.35)	80 (53.33)	
≥ 100,000 copies/mL	125 (45.29)	55 (43.65)	70 (46.67)	
HBsAg positive (n, %)	37 (11.78)	11 (7.80)	26 (15.03)	0.048
Anti-HCV positive (n, %)	13 (4.08)	9 (6.34)	4 (2.26)	0.09
Previous treatment regimens, n (%)				N/A
EFV	158 (49.38)	0 (0)	158 (89.27)	
NVP	19 (5.94)	0 (0)	19 (10.73)	
SQV/r	24 (7.50)	24 (16.78)	0 (0)	
LPV/r	51 (15.94)	51 (35.66)	0 (0)	
ATV/r	62 (19.38)	62 (43.36)	0 (0)	
DRV/r	6 (1.88)	6 (4.20)	0 (0)	
Currently on treatment with TDF (n, %)	305 (95.31)	137 (95.8)	168 (94.92)	0.71
Baseline laboratory				
Chol (mg/dL), median (IQR)	210 (182–239)	205 (182–242)	211 (182–237)	0.90
TG (mg/dL), median (IQR)	138 (94–206)	163 (103–224)	127 (85–192)	0.002
HDL (mg/dL), median (IQR)	46 (38–53)	43 (38–51)	47 (39–55)	0.01
LDL (mg/dL), median (IQR)	131 (107–153)	129 (106–153)	132 (107–154)	0.51
ALT (mg/dL), median (IQR)	26 (19–39)	24 (19–37)	28 (21–41)	0.02
eGFR(CKD-EPI) (mL/min/1.73 m^2^), median (IQR)	101.35 (88.02–109.19)	99.4 (84.1–107.99)	102.62 (92.66–110.72)	0.01
Creatinine (mg/dL), median (IQR)	0.83 (0.72–0.96)	0.84 (0.70–0.97)	0.83 (0.72–0.95)	0.74
Reason for switching to RPV				< 0.001
CNS toxicity	89 (28.25)	3 (2.11)	86 (49.71)	
Simplify regimen	128 (40.63)	108 (76.06)	20 (11.56)	
Dyslipidemia	70 (22.22)	21 (14.79)	49 (28.32)	
Gynecomastia form EFV	5 (1.59)	0 (0)	5 (2.89)	
EFV induce hepatitis	2 (0.63)	0 (0)	2 (1.16)	
LPV/r GI side effect	4 (1.27)	4 (2.82)	0 (0)	
Lipodystrophy	9 (2.86)	0 (0)	9 (5.20)	
ATV induce gall stone	1 (0.32)	1 (0.70)	0 (0)	
Other	7 (2.22)	5 (3.52)	2 (1.16)	

*ART* antiretroviral therapy, *EFV* efavirenz, *NVP* nevirapine, *SQV/r* ritonavir-boosted saquinavir, *LPV/r* ritonavir-boosted lopinavir, *ATV/r* ritonavir-boosted atazanavir, *DVR/r* ritonavir-boosted darunavir, *TDF* tenofovir, *Chol* cholesterol, *TG* triglyceride, *HDL* high-density lipoprotein, *LDL* low-density lipoprotein, *ALT* alanine transaminase, *eGFR* estimated glomerular infiltration rate, *IQR* interquartile range

* P-value by Wilcoxon signed-rank test or t-test

**Fig. 1:**
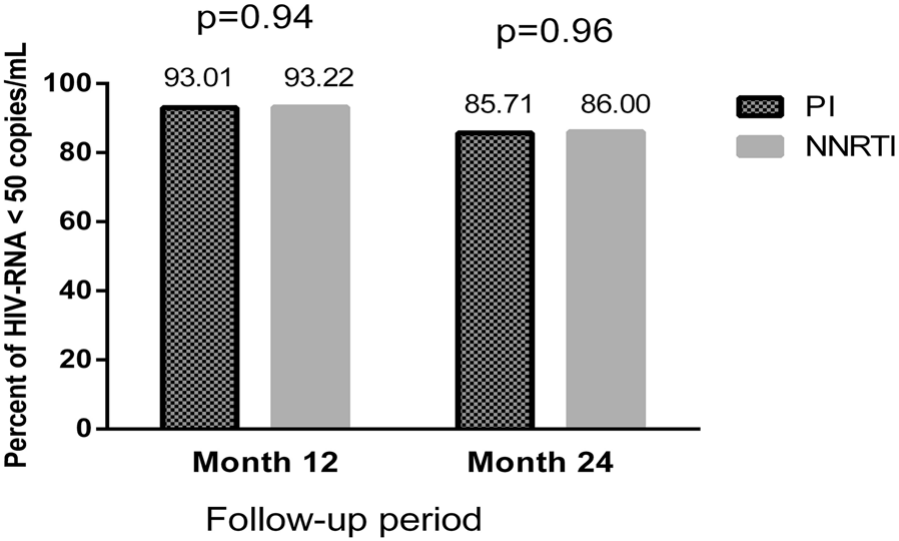
Proportion of patient with a viral load (VL) < 50 copies/mL after 12-months and 24-months follow-up. *PI* protease inhibitor, *NNRTI* non-nucleoside reverse transcriptase inhibitor

After 12 months switching to RPV, 298 (93%) participants maintained virological suppression (HIV RNA < 50 copies/mL). Of 22 participants with unfavorable outcomes, 10 (3.1%) participants discontinued RPV treatment, 7 (2.2%) participants had virological failure, and 5 (1.6%) participants were lost to follow-up during the study period (Fig. 1). There was no difference in virological suppression at month 12 between both NNRTI (165/177 = 93.2%) or boosted PI groups (133/143 = 93.0%; p = 0.94). Discontinuations of therapy occurred for the following reasons: 5 (1.6%) participants had adverse events (AE), 4 (1.5%) participants decided not to continue treatment with RPV-based regimen, and one participant died from coronary heart disease.

Seven participants experienced at least one detectable HIV-RNA up to month 12. Among them, two participants had HIV-RNA > 100 copies/mL and one participant had HIV-RNA > 1000 copies/mL. Treatment interruptions due to AE-related issues were as follows: 2 participants had hepatitis events (Both of them had ALT > 5 times of UNL and returned to normal after discontinuing RPV. No liver biopsy was done), 1 participant had rash, 1 participant had lipodystrophy and 1 participant had QT prolongation. None of the virological failure participants reported any concomitant medication use during the study period.

The median duration of RPV treatment was 1.6 (IQR 1.2–3.0) years. There were 177 participants taking RPV-based regimens for more than 24 months and 86% of overall participants maintained virological suppression to this time point; 85.7% of boosted PI group vs. 86% of NNRTI group (p = 0.96)

**Fig. 2:**
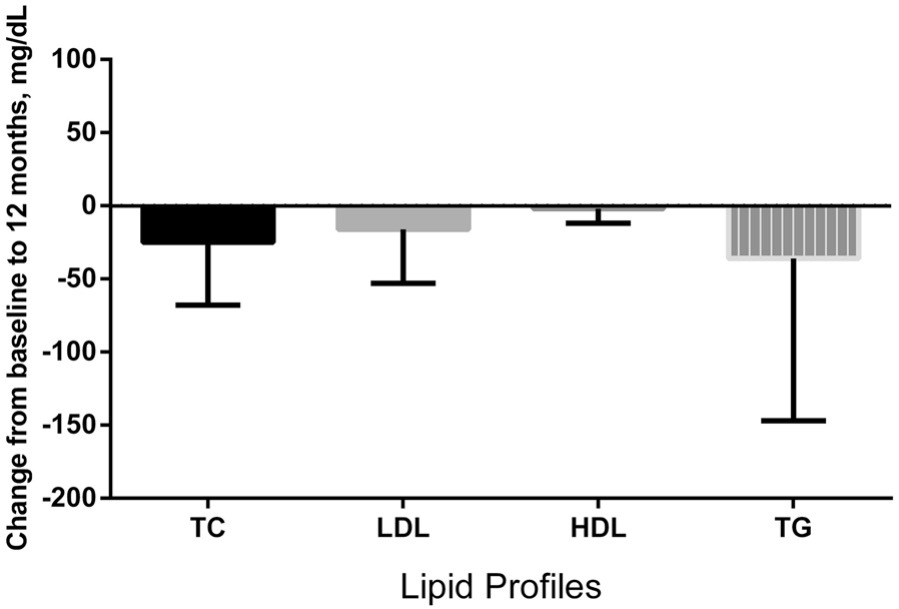
Median changes in lipid profile from baseline to 12 months in patients switching to rilpivirine. *HDL* high-density lipoprotein, *LDL* low-density lipoprotein, *TC* total cholesterol, *TG* triglyceride

Median change in TC, LDL, HDL, and TG from baseline to 12 months were compared (Fig. 2). There were significant improvements in lipid parameters: TC (− 21 (IQR − 47 to 1) mg/dL; p < 0.001), LDL (− 14 (IQR − 37 to 11) mg/dL; p < 0.001) and TG (− 22 (IQR − 74 to 10) mg/dL; p < 0.001). Additionally, the reduction of TG was higher in PI compared to NNRTI-pre-treated groups (− 43 (IQR − 101 to 1) vs − 12 (IQR − 53 to 16) mg/dL; p = 0.002).

We found that estimated glomerular filtration rate (eGFR) had slightly decreased (− 4.3 (IQR − 12 to 1.1) mL/min per 1.73 m^2^; p < 0.001) in overall participants. There was no difference in the reduction of eGFR between PI- and NNRTI-pre-treated groups.

There was a small increase in alanine aminotransferase (ALT) in both groups with median change 5 U/L (IQR − 7 to 14.5; p < 0.001) and the participants in PI-pre-treated groups had increase in ALT more than those in NNRTI group (10 (IQR 2 to 19) VS. 0 (IQR − 12 to 10) U/L; p < 0.001). The median change in cluster of differentiation 4 (CD4) cell count at 12 months was significantly greater among NNRTI pre-treated groups (− 25 (IQR − 125 to 60) VS. 5 (− 78, 93); p = 0.01).

## Discussion

This study demonstrated the efficacy and safety of switching from first line NNRTI- or PI- based to RPV-based regimen among HIV-1-infected Thai participants who had been virologically suppressed with no previous antiretroviral treatment failure. The overall virological suppression at 12 months was 93% among participants who switched to RPV-based regimens, with low rates of virological failure (2.2%) which was similar to previous study reports [14, 15, 17–19]. The randomized SPIRIT study, showed that 89.3% of treatment experienced participants who switched from ritonavir-boosted PI (PI/r)-based regimen to RPV/FTC/TDF were able to maintain viral suppression at week 48 compared to those who continued treatment with a PI/r regimen; indicating a low risk for virological failure [17]. Another study showed that switching from EFV/FTC/TDF to RPV/FTC/TDF a was safe and efficacious option for virologically suppressed HIV-infected participants who cannot tolerate EFV [18].

Only one participant with virological failure had viral load more than 1000 copies/mL and virological suppression was subsequently achieved by reintroducing previous ART. These findings suggest that switching to RPV in routine clinical practice is effective in maintaining virological suppression. Moreover, 86% of the participants remained virologically suppressed at 24 months which is higher than the rate reported by another study (72%) [16].

The main reasons for switching to RPV were toxic AE of the current regimen and simplification of cART. These results are consistent with other studies [14, 19, 20]. We observed a lower rate of RPV treatment discontinuation due to AE in our study compared to a previous study [21] (1.6% vs 7.2%).

Switching to RPV based regimen led to a significant improvement in fasting lipids levels from baseline to 12 months; TC, LDL and TG decreased in both groups but was markedly improved in the boosted PI pre-treated groups. This is in agreement with findings from other studies [15, 19].

Cardiovascular diseases have been recognized as the most common non- acquired immune deficiency syndrome (AIDS) causes of death among HIV-infected individuals on ART [22]. Hypercholesterolemia is known as a major risk factor and requires proper management to reduce cardiovascular disease risk. Therefore, in addition to smoking cessation and other lifestyle modifications, modification of the cART regimen may be additional strategy to reduce cardiovascular risk. However, the median ASCVD risk score did not show statistically significant changes over time in our study.

Regarding renal safety, we found a slightly but statistically significant decrease in eGFR over the follow-up period. RPV is known to cause inhibition of the organic cation transporter in the basolateral membrane of the proximal tubular cell [23]. However, previous data confirmed that participants initially treated with RPV-based regimen had a small decrease in the eGFR that remained stable over the remaining study period [24].

We also found a small but significant increase in ALT values in both groups that was more pronounced in PI pre-treated group. There were two participants who discontinued RPV due to hepatitis (Grade 2–3). This finding is consistent with other studies [19, 21].

Our study has some limitations. First, this is a prospective cohort study in real life setting, so confounding factors could not be completely ruled out. Second, the study populations were only from TRCARC, Bangkok, which may not represent the data from other hospitals in Thailand. Last, we did not have data on concomitant medications (i.e., proton-pump inhibitors), how RPV was taken (i.e., with or without food) and adherence which can interfere with the treatment outcome.

## Conclusion

In many resource-limited settings (RLS), that integrase inhibitors is not affordable, RPV-based regimen would be a good alternative option for PLHIV with first line NNRTI or boosted PI intolerance and without prior NNRTI/PI resistance.

## Abbreviations

AIDS: acquired immune deficiency syndrome
AE: adverse event
ALT: alanine aminotransferase
ART: antiretroviral therapy
cART: combined antiretroviral therapy
CD4: cluster of differentiation 4
EFV: efavirenz
EFV/FTC/TDF: efavirenz/emtricitabine/tenofovir disoproxil fumarate
eGFR: estimated glomerular infiltration rate
FTC: emtricitabine
HDL: high-density lipoprotein
HIV: human immunodeficiency virus
INI: integrase inhibitors
IQR: interquartile range
LDL: low-density lipoprotein cholesterol
LMIC: low- and middle-income countries
NRTI: nucleoside reverse transcriptase inhibitors
NNRTI: nucleoside reverse transcriptase inhibitors
PI: protease inhibitor
PI/r: ritonavir-boosted PI
PLHIV: people living with HIV
RLS: resource-limited settings
RNA: ribonucleic acid
RPV: rilpivirine
RPV/FTC/TDF: rilpivirine/emtricitabine/tenofovir disoproxil fumarate
STR: single tablet regimen
TC: total cholesterol
TDF: tenofovir disoproxil fumarate
TDF + ABC: tenofovir disoproxil fumarate + abacavir
TDF + 3TC: tenofovir disoproxil fumarate + lamivudine
TG: triglycerides
TRC-ARC: Thai Red Cross AIDS Research Centre
